# Duchenne muscular dystrophy in Saudi Arabia: a review of the current literature

**DOI:** 10.3389/fneur.2024.1392274

**Published:** 2024-07-17

**Authors:** Hitham Aldharee

**Affiliations:** Department of Pathology, College of Medicine, Qassim University, Buraidah, Saudi Arabia

**Keywords:** rare disease, genetics, Duchenne muscular dystrophy, DMD, diagnostic delay, awareness, Saudi Arabia

## Abstract

In the past three decades, significant improvements have occurred in the study of Duchenne muscular dystrophy (DMD). DMD is a rare, severe neuromuscular disease that causes death due to cardiovascular and respiratory complications among affected boys. Since the 1980s, ongoing preclinical and clinical studies have been conducted to explore the disease in depth and discover potential therapeutic strategies. In Saudi Arabia, it is unclear whether health services and research efforts are keeping pace with global achievements. Therefore, this review aims to explore the diagnostic and management strategies and research efforts in Saudi Arabia over the past three decades. I searched the PubMed/Medline, Scopus, and Web of Science databases and included all published articles on the epidemiology, genetics, diagnosis, and management of DMD/BMD in this review. The findings suggest a lack of local standardized diagnostic strategies, a poor understanding of epidemiology and common pathogenic variants, and a critical need for preclinical and clinical research. At the time of writing, no such comprehensive review has been published. Challenges, limitations, and future perspectives are also discussed in this article.

## Introduction

Muscular dystrophies (MDs) are genetic diseases caused by pathogenic variants that result in muscle deterioration and loss of function. MDs are classified into nine major groups based on the age of onset, genetic alterations, and clinical presentation (1).

Duchenne muscular dystrophy (DMD; OMIM #310200) is the most common MD and one of the most severe forms (2, 3). The disease is characterized by progressive damage to skeletal and cardiac muscles due to the lack of dystrophin protein (2). Dystrophin is a structural protein that plays a critical role in muscle structure and stability during muscle contraction and relaxation. Dystrophin connects the intracellular cytoskeleton of a myofiber to the extracellular matrix. When dystrophin is absent or nonfunctional, the integrity of the sarcolemma decreases over time, leading to spontaneous muscle degeneration and excessive fat accumulation (4–9).

DMD has an X-linked recessive pattern of inheritance. Thus, DMD affects boys, while girls are mainly asymptomatic carriers, with a chance of experiencing complications (10, 11). Patients with DMD experience difficulties climbing stairs and walking and frequent falls at approximately 2–3 years of age (2, 12). As the disease progresses over time, most patients become wheelchair dependent at approximately 10–12 years of age. Assisted ventilation becomes necessary at approximately 20 years of age (2, 12). Despite significant improvements in patient care, most patients with DMD die at approximately 40 years of age due to cardiac and respiratory failure (13).

The Kingdom of Saudi Arabia (KSA) is the largest country in the Middle East, bordered by the United Arab Emirates, Kuwait, Qatar, Oman, Yemen, the Arabian Gulf, and the Red Sea. The country’s population is estimated at 32 million (14). The Kingdom is divided into thirteen administrative regions, namely Riyadh, Makkah, Madinah, Qassim, Eastern, Tabouk, Aseer, Hail, Northern Borders, Al-Jouf, Najran, Jazan, and Al-Baha.

In KSA, the prevalence of genetic disorders is markedly greater than that in other nations (14). This is attributed to ethnic and social factors, such as high consanguinity rates (14–16). Fortunately, extensive clinical and research programs have been established to better understand and treat common serious genetic disorders. Nevertheless, dystrophinopathies, particularly DMD and BMD, are severely understudied and poorly researched.

This review presents an overview of Duchenne muscular dystrophy in Saudi Arabia, emphasizing the epidemiology, genetic variability, diagnosis, and management of DMD patients. The challenges and research fields in which further investigation is needed are also discussed.

### Healthcare systems and DMD management in Saudi Arabia

The country has mixed public and private healthcare systems. The Ministry of Health (MOH) is currently the major provider and financier of health services. There are more than 484 hospitals across the country. According to Saudi health laws, all Saudi citizens and visitors should be fairly provided with comprehensive and accessible health services. The MOH is responsible for establishing guidelines and strategies for the diagnosis and management of diseases.

Regarding DMD care, the standardization of diagnosis and treatment is considered inadequate and understated. The current diagnostic pathways vary because of the availability of genetic testing and specialized doctors. Patients who live in big cities are most likely to get diagnosed, genetically confirmed with DMD, and treated for complications. In contrast, people who live in small cities or rural areas could be missed and need to be referred to the nearest specialized hospitals for proper diagnosis and treatment. Gratefully, funding for diagnostic tests and treatment is available and supported by the government for Saudis, and by health insurance coverage for non-Saudis.

## Methods

A literature search in the PubMed/Medline, Scopus, and Web of Science database was performed, and all published articles on Duchenne muscular dystrophy in Saudi Arabia since the inception until 2024 were included in this review. The following keywords were used: Duchenne muscular dystrophy (DMD), Becker muscular dystrophy (BMD), muscular dystrophy (MD), Saudi Arabia, epidemiology, diagnosis, and management. Articles were selected according to our search criteria following the PICO strategy: P for population (DMD/BMD), I for intervention (management, diagnosis, and screening), C for comparison to normal subjects, and O for outcomes. The search outcomes included genetic diagnosis, current management, pathogenic variants, and research on DMD/BMD. Only articles published in English and related to DMD/BMD were included in this review. There was no restriction to the type of study to be included in this article. Unpublished data and doctoral and master’s thesis were not included in this review. All the data were extracted, analyzed, and discussed to fulfil the objectives of the present study.

### Epidemiology of DMD

In Saudi Arabia, autosomal recessive disorders are the most common type of genetic disorders (17). Previous reports have shown that approximately 2–5% of all reported monogenic disorders in the KSA are X-linked disorders (18, 19). Moreover, a recent study published by the Centre for Arab Genomic Studies (CAGS) showed that musculoskeletal disorders represent less than 5% of the genetic disorders in the KSA, corresponding to the expected trend of genetic disorders in the Arab population (14).

Globally, DMD affects 1 in 5,000 newborn males (20, 21). The prevalence of the disease is slightly less than 10 cases per 100,000 males (20, 22). On the other hand, the incidence of BMD is 1 in 20,000 male births (10). The incidence and prevalence of DMD have never been reported. It is relatively difficult to determine the incidence and prevalence of DMD/BMD based on the available information; namely, the information is outdated, and there is no updated registry of patients with DMD/BMD. Additionally, at the time of this writing, large-scale epidemiological studies have yet to be published. Therefore, national demographic information is not currently available. Finally, it is highly recommended that a national registry of DMD/BMD patients be established as a keystone for future epidemiological studies as well as treatment and preventive programs.

### Genetic variability among Saudi patients with DMD/BMD

The dystrophin gene (*DMD*; OMIM #300377) is located on the short arm of the X chromosome near the region Xq21 and consists of 79 identified exons, which makes it one of the largest human genes (23, 24). DMD patients exhibit changes in the *DMD* gene, preventing the expression of the muscle isoform of dystrophin (Dp427m) (10, 25, 26). On the other hand, in-frame deletions within the *DMD* gene cause Becker muscular dystrophy (BMD; OMIM #300376), a milder form of DMD (10, 25).

Deletions, duplications, and point mutations (nonsense mutations) are the most predominant mutations detected in patients with DMD (27, 28). Deletions represent 60–70% of *DMD* pathogenic variants, while duplications and point mutations represent 5–10 and 20%, respectively (26, 28, 29). Exons 45–55 and exons 3–9 are known as “hotspot” regions where approximately two-thirds of deletions and duplications leading to DMD/BMD are clustered (26, 28, 29).

The variants patterns within *DMD* have been investigated in different ethnic groups (30–35). Over the last three decades, limited studies on the pathogenic variants of DMD/BMD in Saudi Arabia have been published (Table 1). In 2002, a study showed that deletion of one or more exons was detected in 63% (26 out of 41) of patients with DMD and BMD in the central region of Saudi Arabia (36). A later study conducted in the Western region showed that 62.5% (5 out of 8) of patients with DMD and BMD carried exon deletions within exons 45–55 (37). A subsequent study conducted in the same part of the country reported that deletions were detected in 40% (6 out of 15) of males with DMD, where 20% of the deleted exons contained exon 51 (38). A recent study showed that deletions and duplications represented 46.3 and 53.7%, respectively, of the pathogenic variants in investigated males with DMD (39).

**Table 1:** Summary of the pathogenic variants of DMD/BMD reported in Saudi Arabia.

Author(s)	Year	Study location/site	Sample size	Key findings
Al-Jumah et al. (36)	2002	Central region	41 patients with DMD/BMD	Deletions of one or more exons were found in 26 patients
Chaudhary et al. (37)	2008	Western region	8 unrelated patients with DMD/BMD	Deletions in the distal hotspot region were found in 5 patients.
Taybe (38)	2010	Western region	15 unrelated males with DMD	Deletions of exon 51 (frequency, 20%) and exons 19, 45, and 48 (frequency, 6.7% each) were found in 6 patients.
Elhawary et al. (39)	2018	Western region	15 unrelated male patients with DMD	Deletions: 46.3% (exons 44–56). Duplications: 53.7%.

Although the outcomes of these studies are valuable and consistent with those of DMD/BMD investigations in other populations, robust characterization of *DMD* alterations in the Saudi population still needs to be performed. First, the number of participants reported in the published studies as a percentage of the country’s population is substantially low. Second, most of the available data are limited to patients from the central and western regions, while similar investigations have not been performed in the remaining eleven regions. Third, genetic variations in Saudi female carriers have never been reported. Fourth, genetic variations in exons other than exons 45–55 and exons 3–9 have yet to be screened. This is important because ethnicity and genetic background determine genetic defects in many diseases. In conclusion, regional and national studies incorporating advanced techniques, such as whole-exome sequencing (WES) and Sanger sequencing, are critically needed to elucidate the patterns of DMD among patients and carriers in Saudi Arabia.

### Diagnosis of DMD/BMD

**Figure 1:**
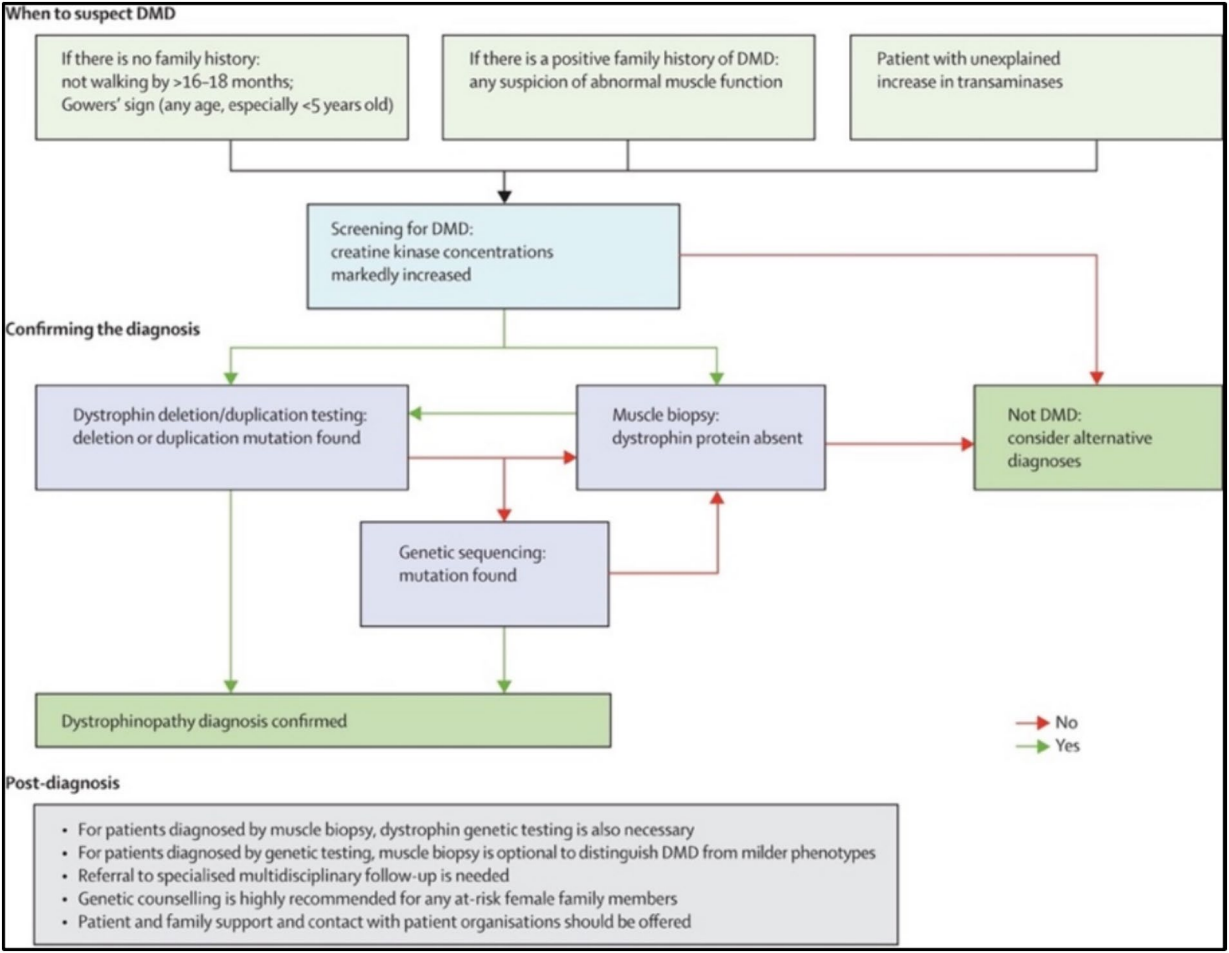
Pathway for diagnosing DMD patients. Reproduced from (41) with permission from Elsevier Ltd. (license number 5584740867112).

Guidelines for diagnosing DMD are available and should be followed (12, 40) (Figure 1). According to published articles about care considerations sponsored by the United States Centers for Disease Control and Prevention (CDC), a diagnosis of DMD should be made as early as possible once signs and symptoms become noticeable, usually at approximately 2–5 years of age (12, 40). The diagnosis process starts with determining the creatinine kinase (CK) level as well as concentrations of other serum enzymes, such as alanine aminotransferase (ALT) and aspartate aminotransferase (AST) (40). Genetic testing is the first confirmatory test for the diagnosis (42). Finally, muscle biopsies for dystrophin staining are recommended for further confirmation for those with clinically compatible dystrophinopathy but with negative genetic findings (11, 12, 43).

The time until intervention and accurate diagnosis significantly impacts effective treatment and prognosis for DMD patients (11, 12, 43). In Saudi Arabia and Middle Eastern countries, a lack of awareness, specific resources, expertise, and standardized diagnostic pathways results in missing the opportunity for early interventions to control the disease (44). Compared with European countries such as the UK, the average delay in DMD diagnosis in Middle Eastern countries is 2–3 years (44). In the UK, the mean age at diagnosis is 4.3 years, while in Middle Eastern countries, the average age at DMD diagnosis is 7–8 years (44). The diagnosis of DMD is also considered late in Middle Eastern countries when compared with Asian countries such as China (5.0 years), Korea (5.0 years), and Taiwan (5.6 years) (45–47).

In Saudi Arabia, a study of the clinical characteristics of Saudi DMD patients revealed a delay in diagnosis due to a lack of awareness of early symptoms (39). The same study showed that 69.8% of participants showed early signs of DMD at 1–3 years of age; however, approximately 44% were diagnosed at 9–12 years of age. Furthermore, a study of four reported cases concluded that the lack of a standardized referral pathway, awareness and specific logistics results in misdiagnosis and delayed diagnosis of DMD in males (16). An observational study of individuals with DMD showed that the mean age at genetic diagnosis of DMD in Saudi Arabia is 6.9 years (42). Unfortunately, there are no standardized referral pathways for DMD patients in the country (44).

In conclusion, the current literature shows that DMD is diagnosed later in Saudi Arabia than in other countries. The country lacks expert clinicians in rare genetic diseases such as DMD. In addition, specialized medical centers for diagnosis and treatment have yet to be established. Patients who live in remote areas of the kingdom face difficulties in accessing specialized health centers, leading to a delayed diagnosis or misdiagnosis of uncommon diseases.

### Screening programs for DMD/BMD

Screening programs for identifying children with DMD/BMD have been suggested. The initial screening test evaluates the serum level of CK. For instance, in the United States, newborn screening for DMD has become mandatory in New York and Ohio, with the goal of expansion to include other states.

Currently, two screening programs exist and are carried out by the Ministry of Health (MOH): premarital and newborn screening programs (48). The newborn screening program was established in 2005 and was expanded in 2016 to include other newborn diseases. However, neuromuscular diseases have not yet been included in the program.

Considering the delay in diagnosis, rate of consanguinity, and lack of awareness, carrier testing could also be a good strategy for early intervention and prevention. Screening for asymptomatic and symptomatic female carriers who have a family history of DMD is seemingly not considered. The identified carriers could be provided with consultation services such as pre-implementation and prenatal genetic testing, improving the health status of the next generation.

### Management of DMD patients in Saudi Arabia

Generally, standardized treatment strategies for DMD patients are relatively insufficient. Currently, there is no cure for the disease, and the primary goal of treatments is delaying disease progression. In Saudi Arabia, glucocorticoids such as prednisone and deflazacort are regularly prescribed to slow muscle degeneration and improve muscle function (11, 44). In addition, management measures for DMD in Saudi Arabia include treating cardiovascular and respiratory complications, gastrointestinal and nutritional complications, and rehabilitation (15, 36).

Although glucocorticoids may not be appropriate for long-term use, newer molecular and genetic therapies have the potential to restore the normal function of dystrophin. The variant-specific therapies ataluren and eteplirsen have already been approved by the European Commission for use within the European Union and the US Food and Drug Authority, respectively (49). Other emerging treatments targeting dystrophin function and normal expression are close to obtaining regulatory approval or have recently been approved (Table 2). Whether these treatments will be available to patients in Saudi Arabia and how those patients will be evaluated and qualify for the therapies are still unknown.

**Table 2:** Updated list of emerging therapies to restore or replace dystrophin.

Therapy	Therapeutic approach	Status
Amondys	Exon skipping	Available to Patients
Elevidys	Gene therapy: micro-dystrophin	Available to Patients
Exondys 51	Exon skipping	Available to Patients
Viltepso	Exon skipping	Available to Patients
Vyondys 53	Exon skipping	Available to Patients
Ataluren	Nonsense mutation readthrough	Phase III
Pf-06939926	Gene therapy: micro-dystrophin	Phase III

*Information listed in the table is adapted from: https://www.parentprojectmd.org/duchenne-drug-development-pipeline/ (Accessed 24 March 2024).

### Patient experience and social support

The management of DMD patients should not be limited to treatment plans and clinical interventions. Management should extend throughout their lives and include their families since the disease is also linked to cognitive abnormalities and social challenges. In general, patients with such diseases are eligible for education and financial and social support in Saudi Arabia. Specialized social workers and social programs are recommended to support patients with untreatable diseases such as DMD.

## Results

A total of 27 articles were found through our search. Nineteen studies that were not relevant to the study topic were excluded. Out of the studies related to DMD/BMD in Saudi Arabia, 4 studies reported the pathogenic variants of DMD/BMD among Saudi patients (Table 1), and one described the demographics and characteristics of DMD patients in the same country (42). One study discussed the management approaches of DMD during the Coronavirus disease 2019 pandemic (15). Another study discussed the current guidelines and approaches of DMD in Middle Eastern countries (44). Finally, a case report of Saudi twin brothers with no family history of DMD was published along with other cases from the Middle East (16).

## Discussion and conclusions

This brief survey of reported studies shows the limitations of DMD research, diagnosis, and clinical interventions in Saudi Arabia. First, there is a substantial lack of basic research and clinical studies focused on the epidemiology and genetic variability of the disease. Moreover, a lack of awareness and social barriers deter families with DMD children from taking the necessary actions for early diagnosis and proper management. Third, the absence of a national or regional registry of patients with DMD is notable. Fourth, the lack of standardized diagnostic approaches results in missing the opportunity for early intervention for the disease and/or misdiagnosis. Fifth, there is a critical need for resources and experts in the health care system and research environment.

**Table:** 

Tips for urgent improvements, establishment of:
Diagnostic referral pathway
Diagnostic algorithm
Family screening and genetic counseling
Tertiary DMD care centers and research institutes

### Recommendations

Despite the challenges in this field, there are potential opportunities for sustainable improvements. The establishment of national and regional research programs is strongly recommended to form a basis for strategic decisions. For instance, funding national screening studies of the prevalence and incidence of DMD in the Saudi population is needed for establishing healthcare facilities and research centers. Moreover, conducting large-scale studies to characterize the genetic variability and common *DMD* alterations in Saudi DMD patients is essential. This knowledge would assist in building a database of standardized treatment strategies and support future clinical trials searching for newer therapeutic approaches.

National efforts to raise awareness of the disease are also essential. Parents and families should be aware of the early signs and symptoms of DMD and how to treat and support affected children and adults. Finally, DMD is still untreatable, and more international collaborative efforts are recommended to offer better support to patients with this disease worldwide.

**Table:** 

Tips for ongoing improvements, programs for:
Updating standard of care
Updating awareness of DMD among medical multispecialty community
Updating awareness of DMD care among patients and families

## Limitations of the review

Due to factors like social barriers and limited communication with the DMD patients or their families, no study has been published detailing their experience or the support they needed from our community. Additionally, the data reported in this article were mainly limited to the central and western regions due to a lack of data from other regions. Finally, published work on DMD/BMD in Saudi Arabia is very limited compared to that in other countries.

## Author contributions

HA: Conceptualization, Data curation, Formal analysis, Funding acquisition, Investigation, Methodology, Project administration, Resources, Software, Supervision, Validation, Visualization, Writing – original draft, Writing – review & editing.
